# Integrating Quadrimalleolar Fractures of the Ankle in a Johannesburg Academic Center: A New Classification

**DOI:** 10.7759/cureus.102048

**Published:** 2026-01-22

**Authors:** Collen Nkosi, Papa Kyei, Brenda Kagodora

**Affiliations:** 1 Department of Orthopaedic Surgery, University of the Witwatersrand, Johannesburg, ZAF; 2 Department of Nuclear Medicine, University of the Witwatersrand, Johannesburg, ZAF

**Keywords:** ankle fractures, complex ankle fractures, new classification, orthopedics, quadrimalleolar fractures

## Abstract

Background

Quadrimalleolar ankle fractures, which consist of trimalleolar fractures with additional anterior fractures, were initially described in a case report. We present a limited series of unusual fractures of the four ankle malleoli and demographic variables to create a comprehensive quadrimalleolar ankle fracture classification system.

Methodology

We conducted a retrospective review of CT scans of patients who sustained ankle fractures at our academic hospital over three years. We included patients who had sustained quadrimalleolar ankle fractures. We classified all fractures based on preoperative CT scans.

Results

A total of 145 CT scans were reviewed, demonstrating quadrimalleolar ankle fractures in just eight cases. Of the eight cases included, 75% were male, and the mean age at the time of injury was 39 years (range = 21-59 years). We integrated quadrimalleaolar ankle fractures into four classifications based on the fracture pattern.

Conclusions

Recognizing these unique fracture patterns as distinct entities would contribute to more comprehensive knowledge and improve the classification system for quadrimalloelar ankle fractures and patient care.

## Introduction

Ankle fractures constitute approximately 5% of all fractures seen in medical facilities in sub-Saharan Africa. In Sub-Saharan Africa, these catastrophic injuries primarily affect young males and result in job losses [1]. Ankle fractures remain among the most prevalent orthopedic injuries on a global scale [2]. Ankle fractures occur at a rate of 168.7 per 100,000 individuals each year over a decade. The majority of ankle fractures that occur are malleolar in origin. Approximately 70% include a solitary malleolus, 20% are bimalleolar fractures, while trimalleolar fractures account for about 10% [3,4].

Quadrimalleolar ankle fractures are rarely reported in the English literature and are anecdotal in occurrence within ankle injuries. Syndesmotic stability is improved by establishing bone-to-bone fixation throughout the anterior and posterior tibial fragments [5]. Rammelt et al. classified fractures of the anterolateral portion of the tibia, also known as the anterior malleolus, into three categories [6]. Posterior malleolar fractures of the ankle are shown to have a prevalence above 40% among ankle fractures [7]. A CT-based categorization method for posterior malleolus fractures, which depends on transverse CT sections, has been described by numerous authors. Mason et al. modified the Haraguchi classification, which provides information on the severity and underlying cause of the fracture [8].

Quadrimalleolar ankle fractures, which consist of trimalleolar fractures with an additional anterior malleolar component, were initially described in a case report in 1964 [4]. For the purposes of this study, quadrimalleolar ankle fractures are defined as fractures involving the anterior malleolus (Chaput fragment), posterior malleolus, medial malleolus, and lateral malleolus. Quadrimalleolar equivalent fractures, involving the Wagstaffe fragment in place of the anterior malleolus, were also included and analyzed within the same classification system.

## Materials and methods

This study retrospectively reviewed 145 adult patients with ankle fractures who underwent CT scanning at our academic hospital between 2020 and 2022. This included patients aged 18 and older and those with quadrimalleolar ankle fractures. We excluded 137 patients who did not meet the inclusion criteria. The study objectives were to determine the demographics of patients with quadrimalleolar ankle fractures at our center and to integrate fracture patterns into a quadrimalleolar ankle classification. Data collection began once the the Univeristy Human Research Ethics Committee (HREC) (Medical) granted ethics clearance (approval number: M240850). We classified all fractures based on radiographs, CT scans, and three-dimensional reconstructions.

Radiological measurements

Plain radiographs were obtained with the patient in a non-weight-bearing posture. These radiographs included anteroposterior, oblique, and lateral views of the ankle joint.

Patient CT scanning

The preoperative CT scans of the patients that involved the ankle were then reviewed. Patients were scanned in the supine position using a multidetector scanner (Siemens SOMATOM Definition AS). The parameters for the CT examination were as follows: kV 120, mA 35, section thickness 1 mm, section collimation 0.6 mm, and reconstruction overlap of 0.5 mm. Patients were scanned by several radiographers in the Department of Radiology at different times of the day.

Assessment of inter- and intra-observer validity

We recruited four additional observers (two registrars and two orthopedic surgeons) to classify the radiographic images of the quadrimalleolar ankle fractures; none of them were involved in the study or patient selection. We trained the reviewers on the new classification system’s workings and provided examples of each type. On two occasions, the four reviewers independently reviewed eight CT scans and plain radiograph images four weeks apart. We randomly organized the eight examples and labeled them with numbers. The cases needed to be shuffled for the same reviewers to re-evaluate them. When recording the categorization findings, the authors noted three categories in succession and a subtype.

Statistical analysis

All data analyses were performed using the Statistica software, version 18.0 (TIBCO Software, Palo Alto, CA, USA). The descriptive statistics for continuous variables were presented as means ± standard deviations, whereas nominal variables were expressed as numbers and percentages. The quadrimalleolar fracture classification systems were analyzed for inter- and intra-observer reliability using the kappa (κ) metric. The κ values were interpreted using Landis and Koch’s standards [9,10]: κ (0-0.20) as slight agreement, 0.21-0.40 as fair agreement, 0.41-0.60 as moderate agreement, 0.61-0.80 as substantial agreement, and 0.81-1 as perfect agreement. Statistical significance was set at a p-value <0.05.

## Results

Demographic characteristics

We reviewed a total of 145 CT scans and found quadrimalleolar ankle fractures in just eight (5.5%) cases. Of the eight cases included, six (75%) were male, and the mean age at the time of injury was 39 years (range = 21-59 years). The cohort commonly suffered right-side ankle injuries at 5 (62.5%), while passenger vehicle accidents were prevalent at 3 (37.5%) in this study (Table 1).

**Table 1:** Demographic characteristics of patients with quadrimalleolar ankle fractures.

	n (%)
Gender	
Male	6 (75%)
Female	2 (25%)
Fracture side	
Left	3 (37.5%)
Right	5 (62.5%)
Mechanism of injury	
*Motorbike accident*	1 (12.5%)
Pedestrian vehicle accident	3 (37.5%)
Motor vehicle accidents	2 (25%)
Fall	2 (25%)
Affected side	
Right	5 (62.5%)
Left	3 (37.5%)
Comorbidities	
Epilepsy retroviral disease	1 (12.5%)
Retroviral disease	1 (12.5%)
Hypertension	1 (12.5%)
None	5 (62.5%)
Social history	
Tobacco user	1 (25%)
Alcohol	1 (25%)
Alcohol and tobacco user	4 (50%)
None	2 (25%)

Anterolateral malleolus fracture-based system

**Figure 1:**
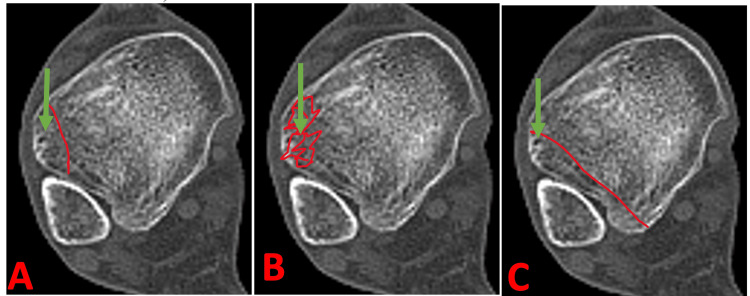
Anterolateral malleolus fracture-based system. (A) Type 1: Small fragment. (B) Type 2: Comminuted fragment. (C) Type 3: Linear fragment with or without mid-fragment comminution. Image source: The Department of Orthopaedic Surgery and Radiology.

**Figure 2:**
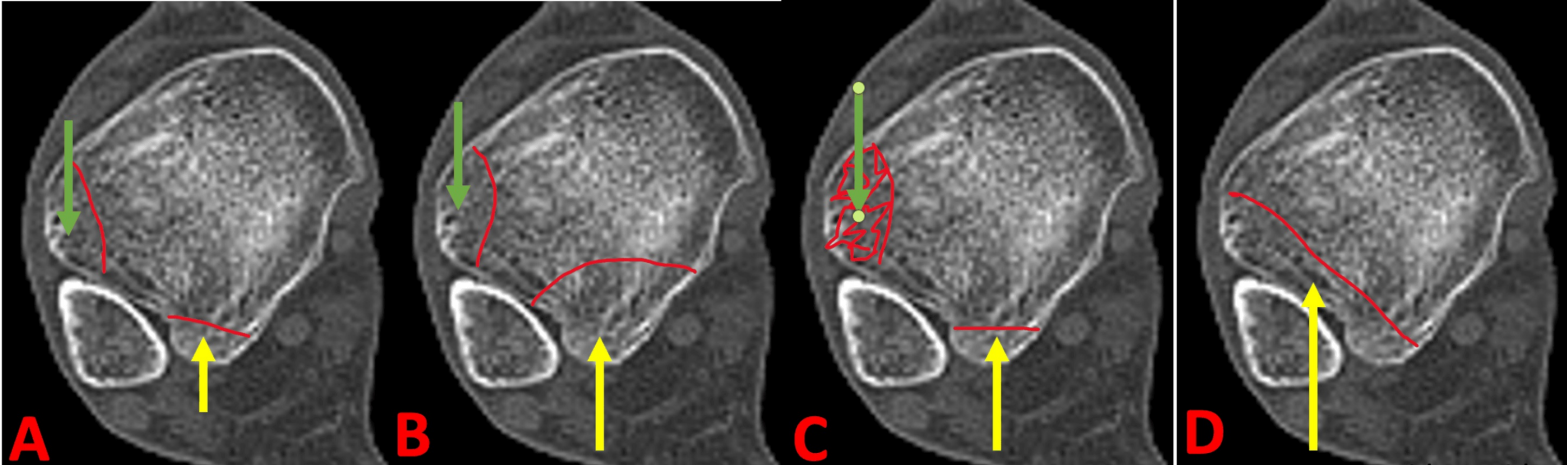
Anterolateral malleolus fractures subtypes. (A) Type 1A: Small fragments of the anterior and posterior malleoli. (B) Type 1B: Malleoli consisting of a small anterior fragment and a large posterior segment. (C) Type 2: Comminuted anterior and small posterior malleoli. (D) Type 3: Linear fragment with or without mid-fragment comminution. Image source: The Department of Orthopaedic Surgery and Radiology.

We identified three distinct anterolateral malleolus fractures: a small fragment, a comminuted fragment, and a linear fragment with or without mid-fragment comminution. We then categorized them into three distinct groups (Figure 1). Of the eight cases identified, four (50%) were type 1, two (25%) were type 2, and two (25%) were type 3 anterior malleolus fractures. We classified the other malleolus fractures as lateral malleolus using the Danis-Weber classification [11], the medial malleolus using the Herscovici classification [11], and the posterior malleolus using the Bartonicek classification [11] (Figure 2, Table 2).

**Table 2:** Characteristics of ankle malleoli fracture categories.

Number of cases	Anterior malleolus: This study	Lateral malleolus: Danis-Weber classification	Medial malleolus: Herscovici classification	Posterior malleolus: Bartoniček classification
Four cases (50%)	Type 1 fragment size	Type A: one case	Type H3: four cases	Type B4: two cases
	-	Type B: two cases	-	Type B2: two cases
	-	Type C: one case	-	-
Two cases (25%)	Type 2: communited fragment	Type C: one case	Type H1: one case	Type B2: two cases
	-	Type B: one case	Type H3:one case	-
Two cases (25%)	Type 3: linear fragment	Type A: one case	Type H4: one case	N/A
	-	Type C: one case	Type H3: one case	-

Proposed new classification system

**Figure 3:**
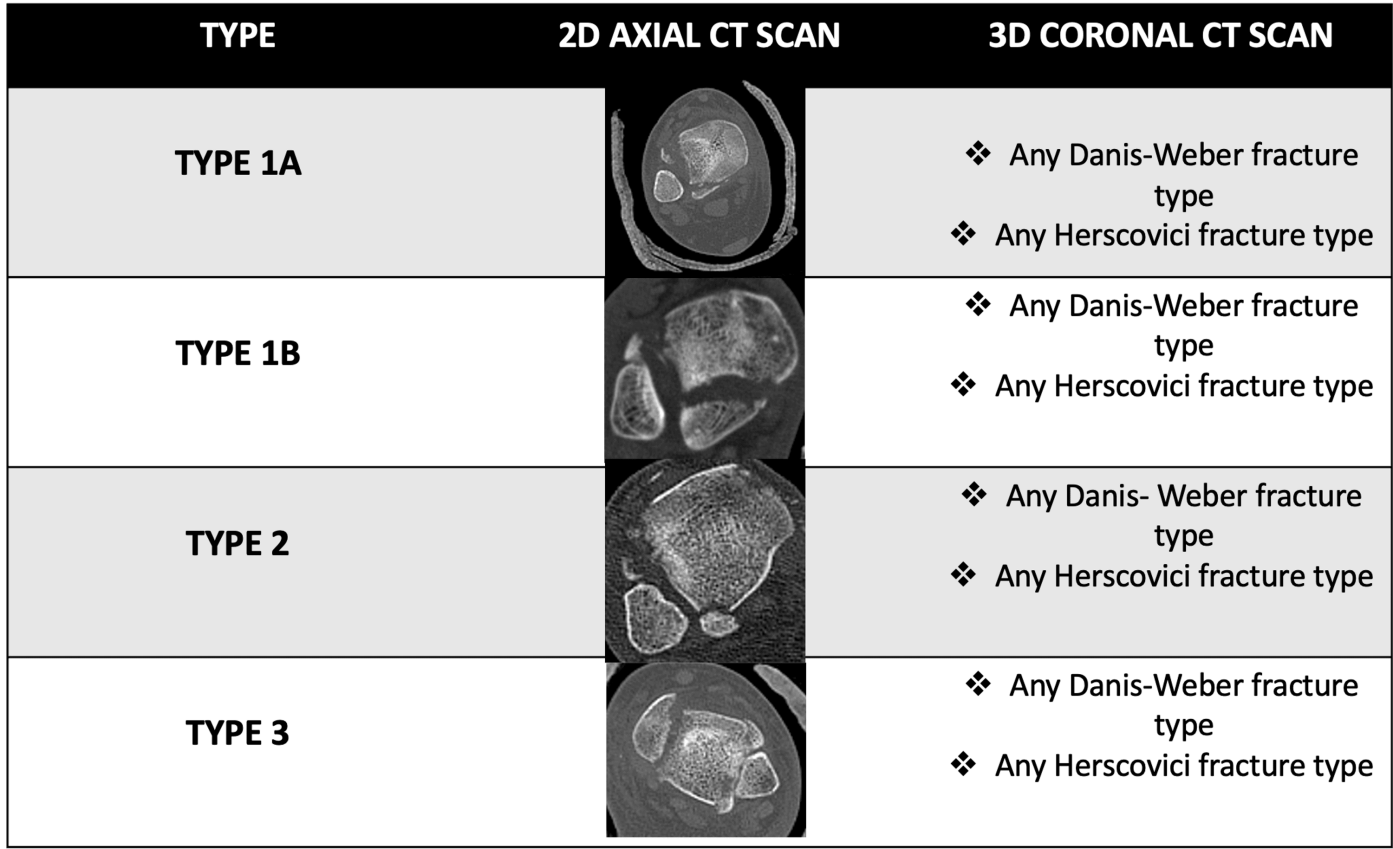
Our proposed quadrimalleolar ankle fractures. Image source: Our Department of Orthopaedic Surgery and Radiology.

Type 1 quadrimalleolar ankle fractures consist of anterolateral fragments associated with any classification of Danis-Weber fractures and any category of Herscovici fractures. This is subclassified into two categories depending on the posterior fragments: type 1A, encompassing posterior malleolus Bartonicek classification type 2, and type 1B, comprising posterior malleolus Bartonicek classification type 4. Type 2 quadrimalleolar ankle fractures are characterized by anterolateral comminuted fragments associated with any classification of Danis-Weber fractures, any category of Herscovici fractures, and a type 2 Bartonicek fracture category. Type 3 quadrimalleolar ankle fractures are characterized by a linear fracture connecting the anterolateral malleolus fragments to the posterior malleolus fragments, associated with any classification of Danis-Weber fractures and any category of Herscovici fractures (Figure 3).

Inter- and intra-observer reliability of the proposed new classification system

The mean κ-value for intra-observer reproducibility, using the new CT-based classification and plain radiographs, was determined to be 0.75 (range = 0.53-1.00), indicating a level of substantial agreement (Table 3). The inter-observer reliability of the new classification system was moderate for both registrars and surgeons on two occasions (Table 4).

**Table 3:** Intra-observer reliability of the new classification system for each repeat observation.

	Agreement %	Kappa statistic	P-value
Registrar 1	75.00%	0.529	0.150
Registrar 2	100.00%	1.000	0.00
Surgeon 1	75.00%	0.680	0.035
Surgeon 2	87.50%	0.781	0.012

**Table 4:** Inter-observer reproducibility of the new classification at different assessments.

	First assessment	P-value	Second assessment	P-value
Registrars and surgeons	0.588	0.032	0.600	0.027
Surgeons	0.461	0.232	0.600	0.091
Registrars	0.600	0.091	0.600	0.435

## Discussion

This study presents a novel classification for quadrimalleolar ankle fractures. The classification is based on the combined fracture morphology of the four malleoli, focusing on radiographs. This classification pertains to anterolateral malleolus ankle fractures, encompassing the lateral, medial, and posterior malleoli [11].

Fractures are classified into three main categories based on the orientation of the fracture morphology in this classification. Type 1 comprises anterolateral fragments associated with any lateral and medial malleolus morphological fracture pattern. Type 1 is subdivided into two types (type 1A and type 1B) based on the posterior fragment size. The size of the posterior fragment determines the subclassification of type 1 into two types, type 1A and type 1B. Type 2 comprises anterolateral comminuted fragments associated with any morphological fracture pattern of the lateral, medial, and small-sized posterior malleolus. Type 3 is characterized by a linear fracture that connects the anterolateral and posterior fragments and any morphological fracture pattern in the lateral and medial malleoli. The novel quadrimalleolar ankle fracture classification system offers a more thorough understanding of fracture anatomy and aids in preoperative fixation planning.

Interesting trimalleolar fractures have been classified as fragility fractures because of their prevalence among the elderly and the gender of the people who sustain them [12]. Low-energy trauma has been described as a prevalent cause of trimalleolar ankle fractures [12]. This study revealed that the average age of the patients was below 40, indicating a predominance of younger male patients with fractures. Overall, 75% of the fractures are associated with traffic accident-related injuries and have a significant social history of smoking and alcohol consumption.

Rammelt et al. reported the surgical procedure and functional outcomes of patients with quadrimalleolar ankle fractures. Their study revealed that restoring the anatomical alignment of the anterior and posterior tibial rim restores the natural contour of the tibial incisura, thereby aiding in the reduction of the fibula [13]. The mean follow-up was 77 months for 79% of the patients. Ankle and hindfoot scale averages of 87, 79, and 15 were recorded for the Foot Function Index, Olerud and Molander Score, and American Orthopaedic Foot and Ankle Society, respectively [13]. Anatomical reduction of quadrimalleolar ankle fractures yields positive functional outcomes.

Kappa (κ) statistics are the standard approach for quantifying inter- and intra-observer variability [9,10]. Our data indicates that the new classification method has significant repeatability and reliability. The mean κ-value for inter-observer reliability suggested “moderate agreement,” measuring 0.588 at the initial assessment and 0.600 in the subsequent evaluation. As observers acquire an understanding of the new classification system, their consistency tends to improve. There was a significant difference in the level of inter-observer reliability between orthopedic surgeons and orthopedic registrars. The second viewing indicated that both surgeons exhibited a comparable mean κ-value. This study demonstrated moderate to perfect intra-observer reliability of the new classification system among orthopedic surgeons and registrars, with a high percentage of agreement.

The study’s limitations include its retrospective design, which introduces potential bias, a small sample size, and the absence of clinical results. This study introduces a unique quadrimalleolar ankle fracture classification system for the first time and confirms its inter- and intra-observer validity, despite its limitations.

## Conclusions

We acknowledge that this study was conducted on a small sample size. It was a retrospective cohort, and based on this limited data, our proposed classification is reliable for characterizing the quadrimalleolar ankle fracture type. We support the use of CT scans as a standard investigation modality and a case-by-case approach to treatment. The classification system has the capacity to delineate fracture patterns and may be employed to assist in the formulation of therapeutic decisions.
